# VennDiagramWeb: a web application for the generation of highly customizable Venn and Euler diagrams

**DOI:** 10.1186/s12859-016-1281-5

**Published:** 2016-10-03

**Authors:** Felix Lam, Christopher M. Lalansingh, Holly E. Babaran, Zhiyuan Wang, Stephenie D. Prokopec, Natalie S. Fox, Paul C. Boutros

**Affiliations:** 1 Informatics and Biocomputing Program, Ontario Institute for Cancer Research, Toronto, Canada; 2 Department of Medical Biophysics, University of Toronto, Toronto, Canada; 3 Department of Pharmacology & Toxicology, University of Toronto, Toronto, Canada

## Abstract

**Background:**

Visualization of data generated by high-throughput, high-dimensionality experiments is rapidly becoming a rate-limiting step in computational biology. There is an ongoing need to quickly develop high-quality visualizations that can be easily customized or incorporated into automated pipelines. This often requires an interface for manual plot modification, rapid cycles of tweaking visualization parameters, and the generation of graphics code. To facilitate this process for the generation of highly-customizable, high-resolution Venn and Euler diagrams, we introduce *VennDiagramWeb*: a web application for the widely used VennDiagram R package. VennDiagramWeb is hosted at http://venndiagram.res.oicr.on.ca/.

**Results:**

*VennDiagramWeb* allows real-time modification of Venn and Euler diagrams, with parameter setting through a web interface and immediate visualization of results. It allows customization of essentially all aspects of figures, but also supports integration into computational pipelines via download of R code. Users can upload data and download figures in a range of formats, and there is exhaustive support documentation.

**Conclusions:**

*VennDiagramWeb* allows the easy creation of Venn and Euler diagrams for computational biologists, and indeed many other fields. Its ability to support real-time graphics changes that are linked to downloadable code that can be integrated into automated pipelines will greatly facilitate the improved visualization of complex datasets. For application support please contact Paul.Boutros@oicr.on.ca.

**Electronic supplementary material:**

The online version of this article (doi:10.1186/s12859-016-1281-5) contains supplementary material, which is available to authorized users.

## Background

Data visualization is a growing and important area of computational biology that demands high quality images which highlight the critical aspects of data. To elucidate all essential features of the data, one must perform a wide range of adjustments to various aspects of the plot, which can be a time-consuming process. Having fine-grained control over the parameters which define colours, fonts, label placements, element sizes, overall resolution, *etc.* leads to more effective plots which can convey necessary details in a publication-ready manner.

Pipelines facilitate automated, robust and reproducible data generation and analysis. Plotting is an important tool for both validation and reporting of results. Incorporating effective plots into these pipelines requires code that has been written specifically for each plot, as there is no single approach which can be applied to varied datasets. As a result, bioinformaticians often engage in long cycles of sequentially modifying plotting code, executing it, and observing the ultimate figure, until an optimum is reached. This process is inefficient and time-consuming.

**Fig. 1:**
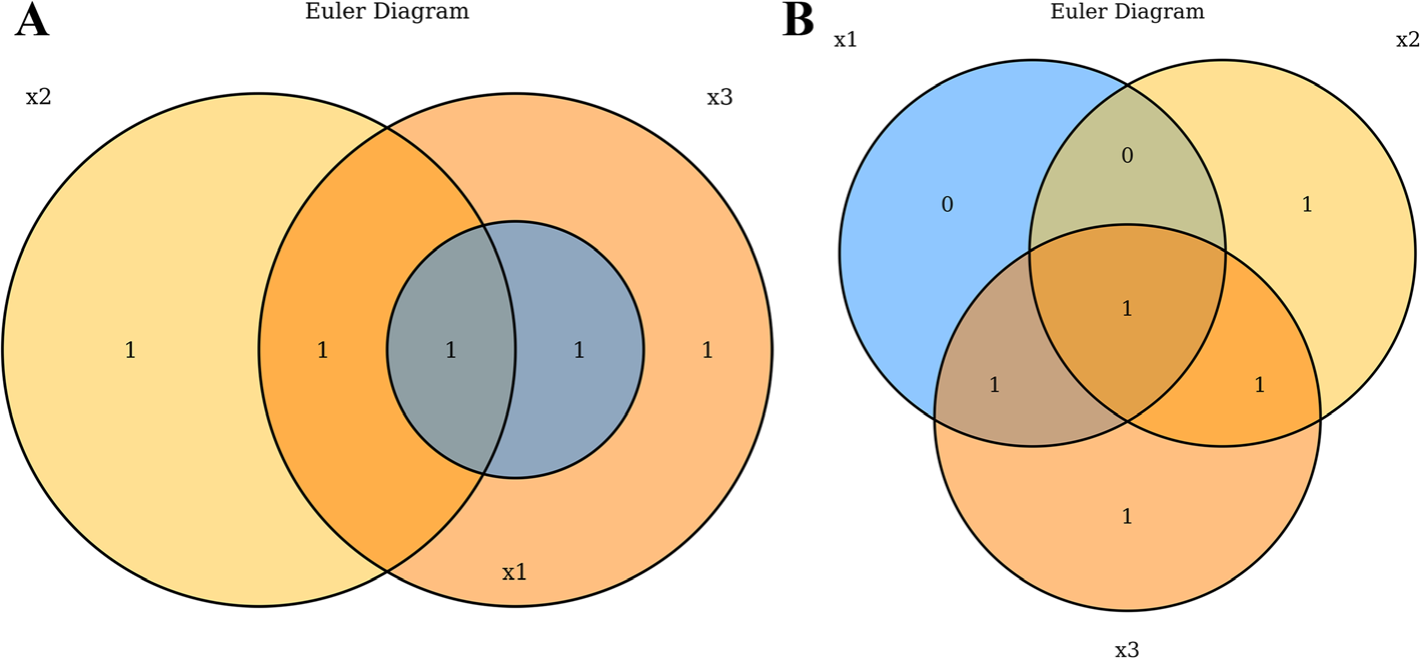
Euler and Venn diagrams produced by *VennDiagramWeb* each depicting three sets: x1 = {7,8}, *x*2 = {4,6,7}, x3 = {4,7,8,10}. **a**. An Euler diagram, produced with euler.d = TRUE and scaled = TRUE. **b**. A Venn diagram, produced with euler.d = FALSE and scaled = FALSE

Venn and Euler diagrams are used frequently in computational biology to visualize the interactions between multiple sets of data. In genomics especially, a common assay is to compare gene lists occurring from separate analyses [1], such as contrasting lists of differentially abundant RNAs following drug treatments or lists of mutated genes across disease types. Venn diagrams are typically depicted as partially intersecting circles or other closed curves such that there are 2^n^ separated regions as depicted by overlapping closed curves [2]. While Venn diagrams always depict all 2^n^ possible regions, Euler diagrams can omit regions under which there are zero values in that region’s subset. This allows Euler diagrams to be less visually complex, by depicting only a subset of all possible regions [3]. *VennDiagramWeb* facilitates the creation of both Venn and Euler diagrams, using the argument euler.d and scaled (both default TRUE). By default, *VennDiagramWeb* will create an Euler diagram where possible, displaying only regions containing one or more values. Users can force Venn diagrams only by setting both euler.d = FALSE and scaled = FALSE (Fig. 1).

The R statistical programming language has widespread use in the bioinformatics field, and so we developed VennDiagram to generate plots in this language [4]. The initial release has proven to be robust and useful, and has garnered 186 citations. As of June 8, 2016 the package has been downloaded from the Comprehensive R Archive Network (CRAN) over 75,000 times since its release in March 2011. Over half of these (>40,000) occurred in 2015 alone, highlighting growing popularity [5].

We believe a graphic user interface for VennDiagram could bring the package to a wider audience and enhance workflows for pipeline developers by providing a real-time framework for plotting optimization. There are many existing web interfaces for creating Venn diagrams, including Venny [6], BioVenn [7], GeneVenn [8], and those from the CRP-Sante Microarray Centre [9] and the Universiteit Gent [10]. These tools perform the necessities of creating a Venn diagram, but are missing many features required to create completely customized publication-quality plots, and have no means of exporting code for integration in large scale analysis pipelines.

## Implementation

**Fig. 2:**
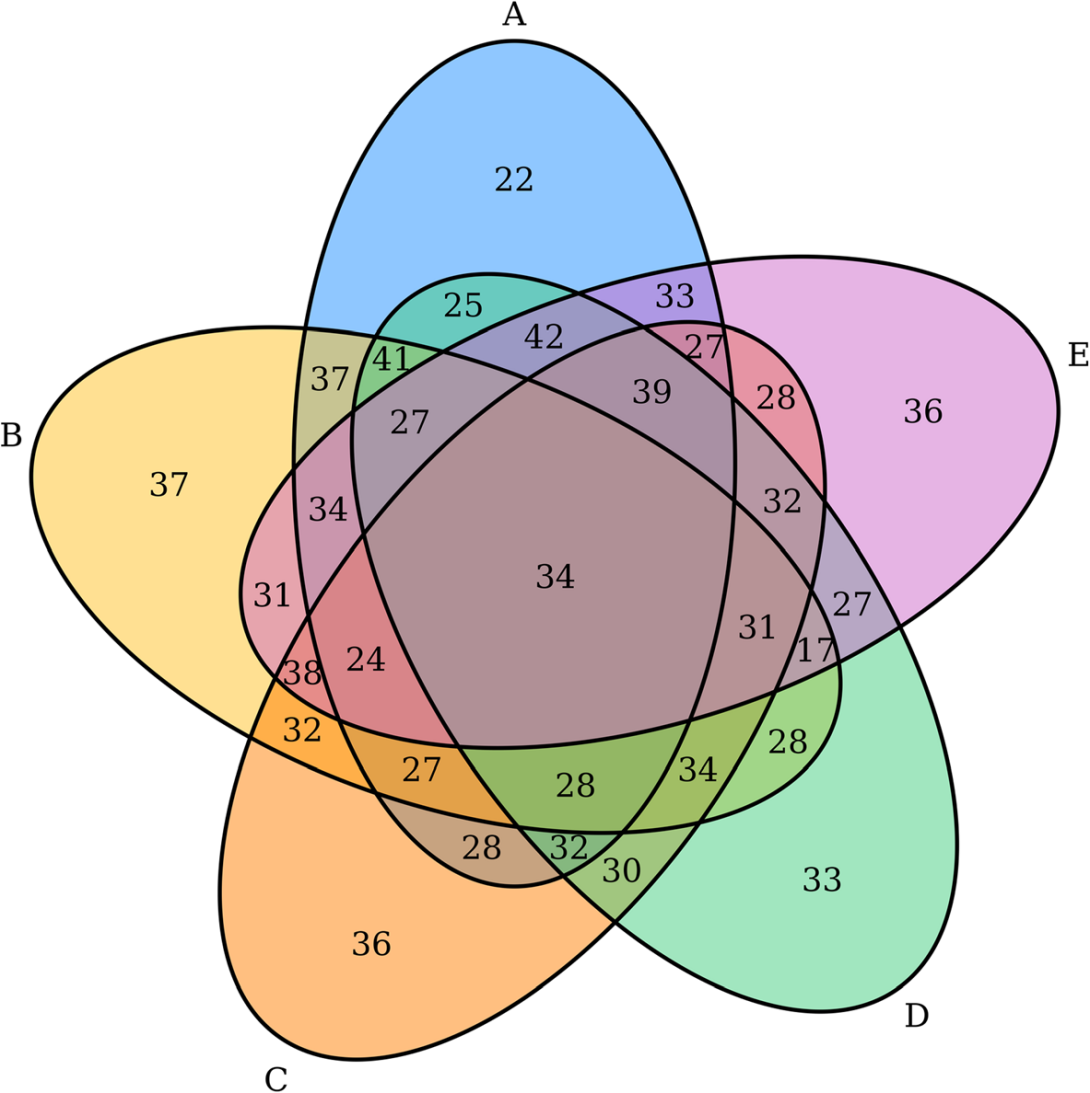
A quintuple set Venn diagram

Our first step was to improve upon the existing VennDiagram R package [4]. A series of changes were made to enhance code quality, including significant refactoring and documentation and exposure of several helper functions. Major feature additions included the ability to create quintuple Venn diagrams. These are highly complex figures, but maintain symmetry and are still easily interpretable (Fig. 2). A parameter to allow users to set a scale by which the areas and labels of the categories will be adjusted to was added. The ability to display proportions of the total population contained within the areas as percentages was also introduced. Many users requested a feature to display a text table of the partitions of the Venn diagram, which is now supported by the package. Users can also now specify an argument which will force the Venn diagram to only consider unique elements in each category when tabulating the sets. In order to have more comprehensive logging which can be integrated with other pipelines which may wrap the Venn diagram code, we now use Futile Logger to log the parameters and sets of the Venn diagrams that are generated at runtime [11]. Finally, users can now choose file types of tiff, png or svg, and can alternatively choose to not output a file, but instead output a list of R graphical objects which compose the entirety of the plot. The user can then modify and re-render the plot as desired.

*VennDiagramWeb* was written using the R statistical programming language and leverages the VennDiagram R package [4]. It uses the web application server Shiny to create a robust graphical user interface which can execute R code on data and parameters dynamically as they change [12]. Using the Shiny web application server enabled us to create a solution that is composed nearly purely of R from end to end. Using a single language allows for very tight integration of security and error handling functionality. We are able to parse any inputs provided by the user and, using functionality built into the language, inspect those inputs to ensure there is no attempt to inject malicious code. The architecture of the web application is based around the code for the user interface and the code for the server. The user interface is defined by a series of widgets which accept the parameters and data files from the user, and display the rendered plot reactively as elements are changed. The server handles all arguments and data, ensures that they are safe and valid, and performs generation of figures.

## Results

### User interface

**Fig. 3:**
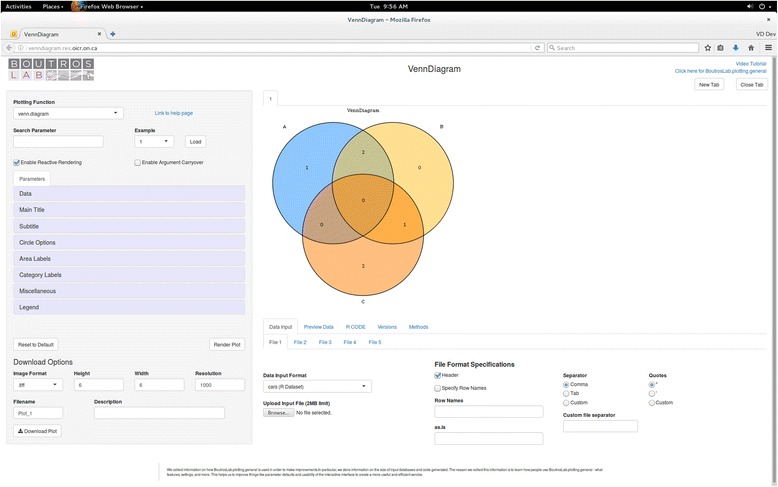
The *VennDiagramWeb* user interface

**Fig. 4:**
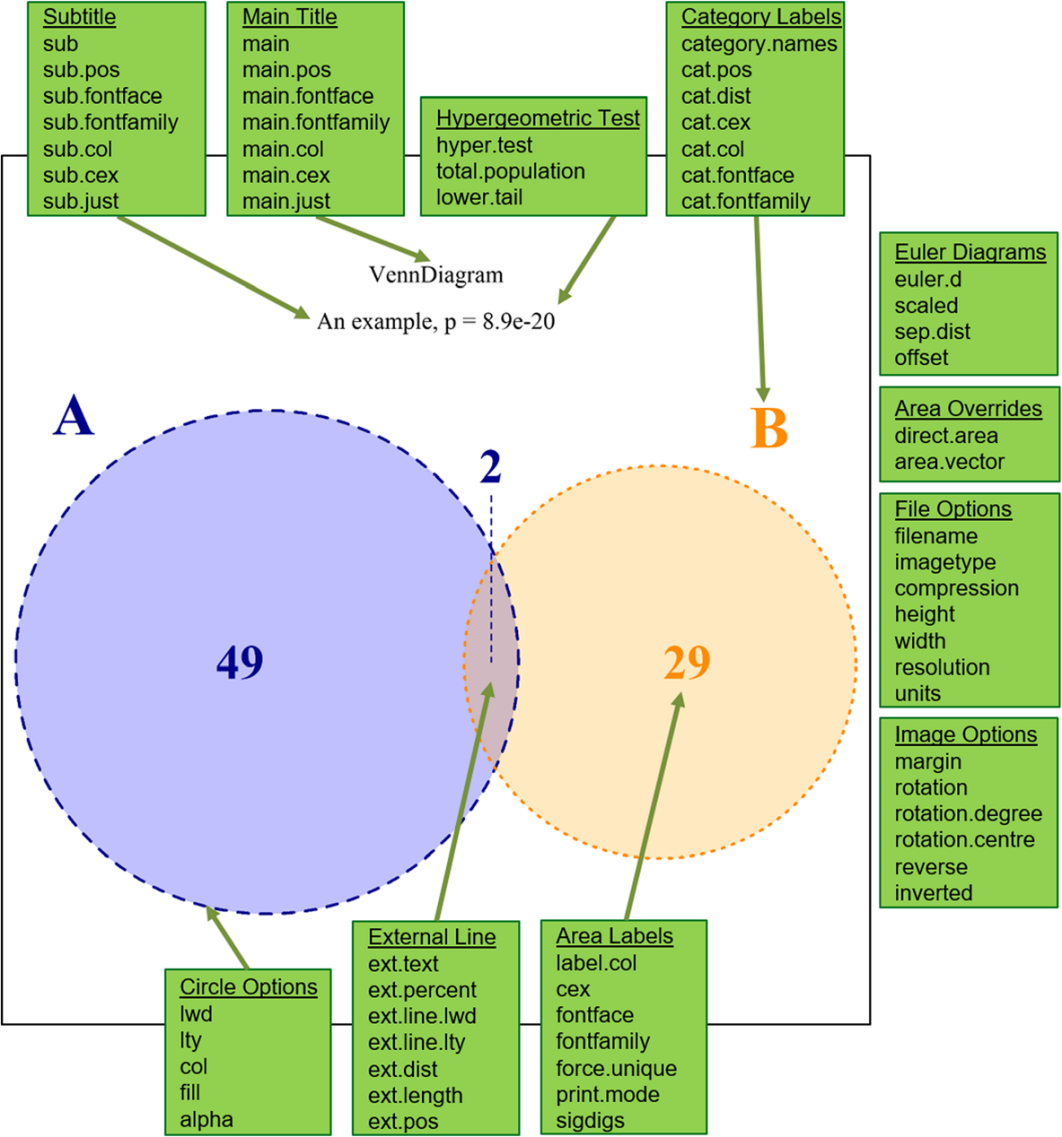
A Venn diagram generated using *VennDiagramWeb*, annotated to indicate the parameters corresponding to features of the plot. The panels in *green highlight* the parameters of the tool, showing with arrows what elements of the Venn diagram are directly affected. All green elements are not generated as part of the Venn diagram

*VennDiagramWeb* is a graphical user interface for the venn.diagram function [13]. The application starts with a simple example loaded (Fig. 3). Users can also choose to load an example configuration using the drop-down menu in the top right area of the sidebar. Users can modify the parameters of the venn.diagram function using the sidebar, and the resultant plot is generated instantly in the center panel (Fig. 4). The parameters for venn.diagram are divided into eleven sections, allowing the user to quickly find parameters of interest. If the user is familiar with the R package VennDiagram, they can also search for a parameter by name. At the bottom of the sidebar, the user can download the plot displayed as an image. On the bottom bar, the user can choose the datasets plotted, preview the datasets and data partitions, view the R code used to generate the plot, and access proper citation information for *VennDiagramWeb*.

### Features

*VennDiagramWeb* is meant to integrate seamlessly into scientific plot generation workflows. To this end we have included several key features: data file uploads, access to underlying R code, image downloads, and multiple workspaces.

#### Data file uploads

Users can upload up to five data files, up to 2 megabytes each, for use in generating their diagrams, which is quite large for Venn diagrams. These datasets are made available to the editor as dataframes titled data1, data2, *etc.* through to data5. This feature is found in the first tab on the bottom bar. The file uploading system is designed to accept tables output by R using the write.table function, but can accept any simple text table format, such as csv, tsv, and txt. Some options are available for file format customization, generally pertaining to the options of write.table. These options allow the user to specify the column separator, the presence of headers and/or row names, and whether or not values are contained in quotes. The user is able to preview the first four lines of the datasets available. As an example, we have provided sample data for the reader to upload (Additional file 1) and visualize (Additional file 2) [14].

#### R code access

As *VennDiagramWeb* is a graphic overlay for the R package VennDiagram, the user can access the generated R code to reproduce the plot as it appears on screen. This is useful because it allows the user to rapidly prototype the appearance of their desired Venn diagram, and then download the corresponding code for integration into their own pipelines. It is also a necessary feature because though a web application is user-friendly, many users may wish to avoid uploading their data to an external website, or may be restricted from doing so by privacy laws [15]. For this reason, users can experiment with *VennDiagramWeb* for diagram formatting and customization on toy datasets, and then download the resulting code for use with their real data.

#### Image downloads

*VennDiagramWeb* allows users to download publication-quality images of the Venn diagram displayed on screen. Users can choose the image format as tiff, png or svg, as well as resolution and physical size in inches.

#### Multiple workspaces

The web application has an interface for creating and switching between tabs. This allows users to create several different plots simultaneously. Each workspace tab is distinct and does not share data or parameters to avoid unintentional effects on diagrams which are being generated concurrently.

## Discussion

### Benefits

The datasets analyzed in biology and particularly in genomics can be enormous in scope and complexity, and we can only expect them to grow [16]. The rise of big data has lead to increasing attention to the field of data visualization [17]. As our datasets increase in size and our analyses increase in complexity, data visualization becomes crucial in allowing us to gain insight, see patterns and elucidate further areas of study in our experiments [17–19]. These visualizations have to be meaningful representations of the data [18]. Unsurprisingly, the figures we use to communicate our insights can become more convoluted with bigger and more complex data and, if poorly designed, risk confusing what they were meant to make clear. Indeed, the difficulty of achieving visual clarity in a diagram often increases in tandem with the necessity to do so.

Despite the growing importance of data visualization, the scientific community faces technical challenges in creating figures that are meaningful and clear. Complex analyses and their visualizations can represent significant time and effort, and require programming skills the scientific community often lacks [20]. A paper with poorly designed figures could be significantly hampered: if a reader cannot grasp the key insights a figure is meant to convey, then the significance of the paper might be lost, or it may not be published at all, with obvious detriments to the field. There is a clear incentive to generate useful plots, and there is also a clear need for easier and more time-efficient methods to generate them. To this end, we are creating tools that improve both the quality of plots and the speed at which they can be created.

In genomics, a common analysis performed in experiments is list comparison. The Venn Diagram is a popular plot type for list comparison, providing a simple visualization of several possibly unrelated lists [1]. The manual generation of these plots can be difficult. Spatial organization and colour choice of the groups and their overlapping sections such that they are both clear and aesthetically pleasing is important but nontrivial [21]. The VennDiagram R package addresses this issue. It is commonly in use [5], but we believe a simple graphical user interface for the package will bring it to an even wider audience, particularly to scientists who do not have strong skills in R.

### Comparison to existing tools

As with VennDiagram, *VennDiagramWeb* offers many features that existing tools lack (Table 1). In addition to the advantages VennDiagram holds over other methods [4], *VennDiagramWeb* is the only web application we found that allows for tiff downloads, R code download, and multiple workspaces.

**Table 1:** A comparison of features of *VennDiagramWeb* to other popular web applications used to generate Venn Diagrams

Category	Parameter	VennDiagram (Initial Release)	VennDiagramWeb	Venny [3]	BioVenn [4]	GeneVenn [5]	CRP-Sante Microarray Centre Venn Diagram [6]	VIB/UGent Calculate and draw custom Venn diagrams [7]
General	Interative Interface		X	X	X	X	X	X
	Data File Upload	N/A	X		X	X		X
	Multiple Workspaces	N/A	X					
	R Code Generation	N/A	X					
Circle Options	Colour	X	X		X	X		
	Fill Alpha	X	X					
	Border Style	X	X					
	Border Width	X	X					
	Border Colour	X	X					
Category Labels	Content	X	X			X		
	Colour	X	X		X			
	Font	X	X		X	X		
	Size	X	X		X	X		
	Style	X	X					
	Location	X	X		X (SVG only)			
	Position	X	X		X (SVG only)			
	Distance	X	X		X (SVG only)			
	Justification	X	X					
Area Labels	Colour	X	X		X			
	Font	X	X	X	X	X		
	Size	X	X	X	X	X		
	Style	X	X					
Titles	Main title	X	X		X	X		
	Subtitle	X	X		X			
	Position	X	X		X (SVG only)			
	Colour	X	X		X			
	Font	X	X		X			
	Size	X	X		X			
	Style	X	X					
	Justification	X	X					
File Options	Output type	TIFF	TIFF/SVG/PNG	PNG	SVG/PNG	PNG	PNG	SVG/PNG
	Figure resolution	X	X		X			
	Built-in gene ID recognition				X			
Miscellaneous	Maximum sets	4	5	4	3	3	3	3
	Shapes used	Circles/Ellipses	Circles/Ellipses	Circles/Ellipses	Circles	Circles	Circles	Circles/Ellipses/other curved shapes
	Scaling		X^b^		X^a^			
	Euler diagrams	X	X		X			
	Margin size		X					
	Rotation		X					
	Two-set external lines		X					
	Other set-specific parameters		X					
	Partition display		X	X	X	X	X	X

^a^Uses inaccurate 3-set scaling with circles

^b^Euler diagrams only. 3-set scaling only when mathematically possible [4]

## Conclusions

*VennDiagramWeb* provides a unique way to integrate and optimize R code for data-visualization with web-based real-time manual optimization with computational pipelines. It will be a key resource for the field. For application support please contact Paul.Boutros@oicr.on.ca.

## Additional files

Additional file 1: Table S1. Sample data for the user to upload. Hypergeometric testing was conducted on samples of rat and mouse liver tissue to identify similar transcriptomic profiles [14]. (XLS 189 kb) Additional file 2: Figure S1. Venn Diagram created in *VennDiagramWeb* using Supplemental Table 1 [14] uploaded as data1, with x1 = data1[data1$Mouse.Liver.Q < 0.05,]$HomologeneID. *x*2 = data1[data1$Rat.Liver.Q < 0.05,]$HomologeneID. x3 = NULL. category.names = Mouse,Rat. cat.pos = 180. cat.dist = 0.02 and all other arguments default. (TIFF 758 kb)

## Abbreviations

CRAN: Comprehensive R Archive Network
