# Supplementary figures and images for: VennDiagramWeb: a web application for the generation of highly customizable Venn and Euler diagrams
Source: BMC Bioinformatics. 2016 Oct 3;17:401. doi: 10.1186/s12859-016-1281-5 (PMC5048655; doi:10.1186/s12859-016-1281-5)

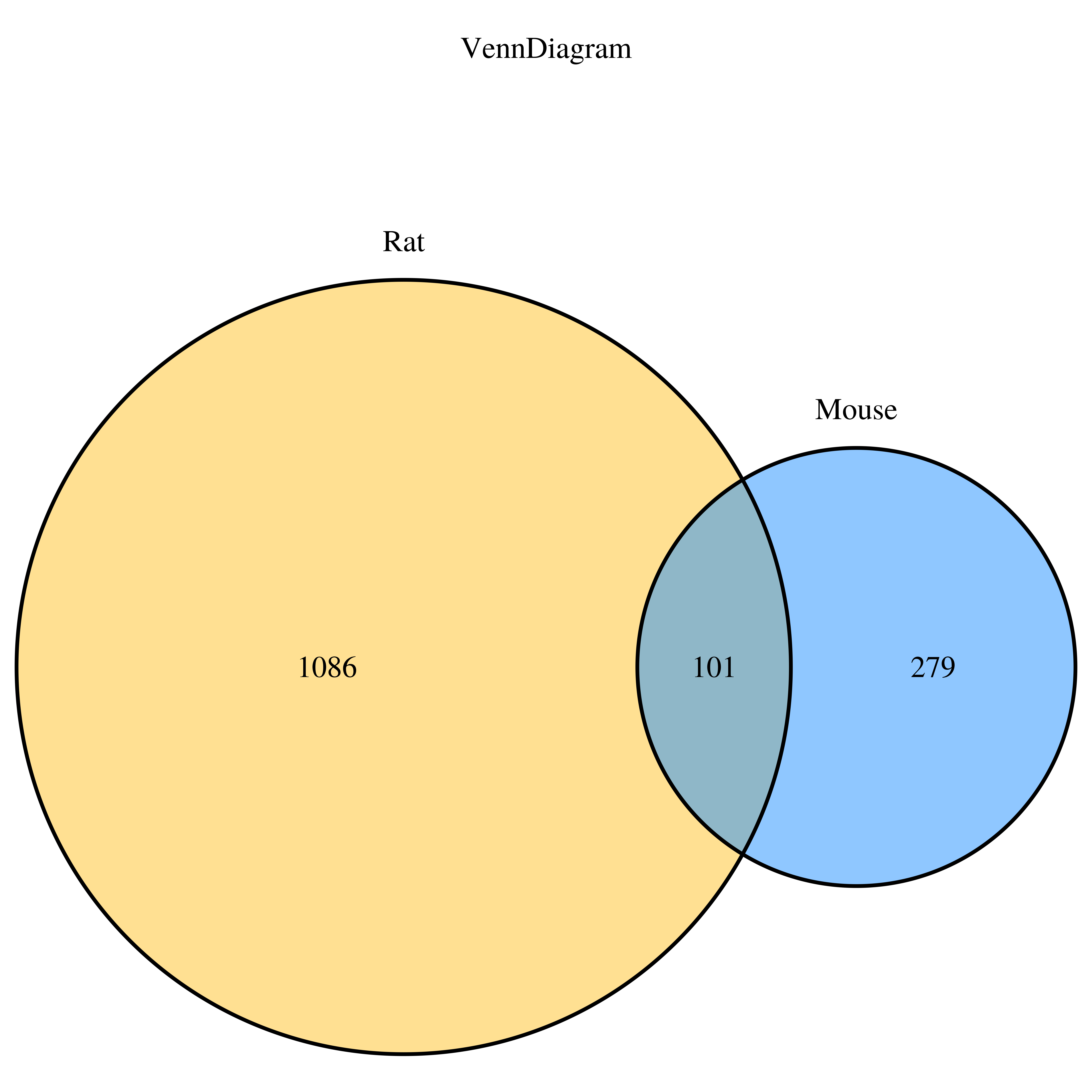

Supplement: Additional file 2: Figure S1. — Venn Diagram created in VennDiagramWeb using Supplemental Table 1 [14] uploaded as data1, with x1 = data1[data1$Mouse.Liver.Q < 0.05,]$HomologeneID. x2 = data1[data1$Rat.Liver.Q < 0.05,]$HomologeneID. x3 = NULL. category.names = Mouse,Rat. cat.pos = 180. cat.dist = 0.02 and all other arguments default. (TIFF 758 kb) [file 12859_2016_1281_MOESM2_ESM.tiff]
